# Indigenous Yeasts for the Biocontrol of *Botrytis cinerea* on Table Grapes in Chile

**DOI:** 10.3390/jof9050557

**Published:** 2023-05-11

**Authors:** Ximena Sepúlveda, Marisol Vargas, Silvana Vero, Nelson Zapata

**Affiliations:** 1 Facultad de Agronomía, Universidad de Concepción, Avenida Vicente Méndez 595, Chillán 3780000, Chile; xsepulveda@udec.cl (X.S.); nzapata@udec.cl (N.Z.); 2 Facultad de Química, Universidad de la República, Av. Gral. Flores 2124, Montevideo 11800, Uruguay; svero@fq.edu.uy

**Keywords:** antagonistic yeast, gray mold, modes of action

## Abstract

One hundred twenty-five yeast strains isolated from table grapes and apples were evaluated for the control *Botrytis cinerea* of in vitro and in vivo. Ten strains were selected for their ability to inhibit mycelial growth of *B. cinerea* in vitro. In the in vivo assays, these yeasts were tested at 20 °C on ‘Thompson Seedless’ berries for 7 days; only three were selected (m11, me99 and ca80) because they significantly reduced the incidence of gray mold. These three yeast strains were then evaluated at different concentrations (1 × 10^7^, 1 × 10^8^ and 1 × 10^9^ cells mL^−1^) on ‘Thompson Seedless’ grape berries at 20 °C. The strains m11, me99 and ca80 reduced the incidence of *B. cinerea* to 11.9, 26.1 and 32.1%, respectively, when the berries were submerged in a yeast suspension at a concentration of 1 × 10^9^ cells mL^−1^ 24 h before inoculation with *B. cinerea*. The most favorable pH for antifungal activity was 4.6 in the three isolates. The three yeast strains secreted the hydrolytic enzymes chitinase and β-1-glucanase, and two strains (me99 and ca80) produced siderophores. The three yeast strains exhibited low oxidative stress tolerance and only strain m11 had the ability to produce biofilms. The strains were identified using 5.8S-ITS rDNA PCR-RFLP and correspond to the *Meyerozyma guilliermondii* (m11) and *Aureobasidium pullulans* (me99 and ca80) species.

## 1. Introduction

*Botrytis cinerea*, the causal agent of gray mold, is the main phytosanitary problem for table grapes in Chile because it limits table grape production and export [1]. This species hibernates as a saprophyte in necrotic tissues or as sclerotia or mycelium on tissues [2]. From these fungal structures, *B. cinerea* easily releases its conidia when the climate is humid [3]. Conidia can infect the vine during different developmental stages, being most vulnerable between veraison and harvest, due to the particular vulnerability of the berries [4].

Control of this pathogen is mainly exercised by applying fungicides and sanitary measures at the most critical periods for infection in order to maintain a low rate of damage and extend the postharvest life of the fruit [5]. However, growing public concern about fungicidal residues in food, the environmental risk associated with their use in the orchard, and the development of resistant strains of pathogens to these fungicides in Chile [6,7,8,9,10] has generated interest in the development of alternative non-chemical control methods, with biological control as one of the most promising and explored alternatives [11].

The use of antagonistic microorganisms has been recognized as one promising alternatives to applying fungicides [12]. Among these microorganisms, antagonistic yeasts isolated from the surfaces of fruits have been evidenced to effectively control postharvest diseases [13,14,15,16] since they have some useful properties, such as the ability to colonize the surface of fruits for long periods of time under drought conditions, production of extracellular polysaccharides that enhance their ability to survive, rapid use of available nutrients, and being minimally affected by pesticides [17].

A good biocontrol agent requires multiple modes of action to antagonize a pathogen [18]. Among the reported modes of action in yeasts are competition for nutrients and space, parasitism, secretion of hydrolytic enzymes, siderophore production, production of volatile organic compounds, ability to produce biofilm, and the induction of resistance [15,19,20,21,22,23,24,25].

Yeast biocontrol agents include *A. pullulans*, which reduced gray mold on kiwifruit [26]; *Candida oleophila*, which reduced gray mold and black rot on kiwifruit [27]; *Geotrichum candidum*, which reduced blue mold on grapes [28]; *Kloeckera apiculata*, which reduced blue mold on apples [29]; *Metschnikowia pulcherrima*, which reduced gray mold on apples [30]; *Pichia membranefaciens*, which reduced Rhizopus rot on peaches [15]; *Sporidiobolus pararoseus*, which reduced gray mold on strawberries [31]; and *Wickerhamomyces anomalus*, which reduced brown rot on peaches and plums [32]. As a result of these studies, some commercial formulations based on yeast have been developed, such as Blossom Protect, Botector (*A. pullulans*), Nexy (*C. oleophila*), Noli, Shemer (*Metschnikowia fructicola*) and Remeo (*Saccharomyces cerevisiae*) [33].

In this research, we selected and characterized indigenous epiphytic yeasts for the biocontrol of *B. cinerea* in table grape berries, and we determined the mode(s) of action for the yeast strains with the most auspicious biocontrol activities against the pathogen.

## 2. Materials and Methods

### 2.1. Fruit

Table grape berries (*Vitis vinifera*) “Thompson Seedless” from a commercial orchard located in San Francisco de Mostazal (Cachapoal Province, Chile) were used in this study. The berries were superficially disinfected with 0.5% sodium hypochlorite for 3 min, rinsed three times with distilled water, and dried in a laminar flow chamber.

### 2.2. Pathogen

A *Botrytis cinerea* isolate was isolated for Vargas et al. [34], available from the microorganism collection at the Phytopathology Laboratory of the Faculty of Agronomy at the Universidad de Concepción, and maintained on potato dextrose agar (PDA) at 4 °C.

### 2.3. Antagonists

Yeasts were isolated from the surfaces of berries from different grape cultivars (‘Dawn’, ‘Cabernet Sauvignon’, ‘Merlot’, ‘Moscatel de Alejandría’, ‘País’, and ‘Thompson Seedless’) and healthy apples (‘Royal Gala’) from commercial orchards in Chile. The fruit was washed by dipping in sterile saline solution (0.9% NaCl), then the obtained suspension was diluted (1:10) and 100 μL was spread on Yeast Peptone Dextrose Agar (YPD) at pH 4.6, followed by the addition of 0.05 g L^−1^ of streptomycin (Sigma-Aldrich, St. Louis, MO, USA) and chloramphenicol (Sigma-Aldrich, St. Louis, MO, USA). The petri dishes were incubated at 25 °C to observe the development of colonies [35]. The single-cell yeast colonies were re-cultured in YPD and stored at −20 °C in cell suspension with 30% *v*/*v* of glycerol and 70% YPD broth.

### 2.4. Selecting Yeasts with Antagonistic Activity against Botrytis cinerea

#### 2.4.1. Inhibition of Mycelial Growth of *Botrytis cinerea*

Every yeast isolate was co-cultured with the pathogen in Petri dishes containing Malt Extract Agar (MEA). A 5 mm mycelial disc, obtained from the edges of a 5-day-old culture of the fungus, was placed in the center of the dish. Four isolates per plate were streaked, one in each quadrant, and the plates were incubated at 25 °C for 7 days. The level of mycelial growth inhibition was determined by the Swadling and Jeffries [36] scale: 0 = without any visible signs of *B. cinerea* inhibition, mycelium surpasses antagonist colony; 1 = both organisms stop growing on contact; 2 = inhibition zone between pathogen and antagonist is <2 mm; 3 = inhibition zone is between 2 and 4 mm; and 4 = inhibition zone is >4 mm. Yeast strains that attained a value between 2 and 4 in this scale were selected for the next bioassay.

#### 2.4.2. Inhibition of Gray Mold Rot on Table Grape Berries

The isolates that inhibited the mycelial growth of *B. cinerea* were evaluated for biocontrol activity. Berries were wounded along the equatorial axis and submerged in a yeast suspension at a concentration of 1 × 10^8^ cells mL^−1^. After 2 or 24 h, the fruit were sprayed with a suspension of pathogen conidia at a concentration of 1 × 10^4^ spores mL^−1^. Each selected yeast strain was considered a treatment, and these treatments were established in a completely randomized design with three replicates, with each replicate made up of 28 berries. Rot incidence was recorded after 7 days of storage at 20 °C, according to Vero et al. [37].

### 2.5. Identifying Yeasts with Biocontrol Potential against Botrytis cinerea

The identification of yeast strains, selected for their ability to biocontrol grey mold, was performed using 5.8S-ITS rDNA PCR-RFLP. The DNA was extracted from pure cultures with a DNeasy Plant mini Kit (Qiagen) according to manufacturer instructions. PCR was carried out using a ITS1 primer (5′ TCCGTAGGTGAACCTGCGG 3′) and a ITS4 reverse primer (5′ TCCTCCGCTTATTGATATGC 3′). The amplification reaction was performed under the following conditions: 7 min at 95 °C; 35 cycles at 94 °C for 1 min, 30 s at 55.5 °C, and 1 min at 72 °C followed by a final step of 10 min at 72 °C. The PCR products were digested with restriction endonucleases *HhaI*, *HaeIII* and *HinfI* (New England Biolabs Inc., Beverly, MS, USA) according to manufacturer instructions [38]. The PCR products and the restriction fragments were analyzed with electrophoresis in 3% agarose gel. Strain identification was confirmed by sequencing of the 5.8S-ITS region of the ribosomal RNA by Macrogen Inc. (Seoul, Republic of Korea). The sequences were compared with those published in the GenBank database with the BLAST program.

Phylogenetic analyses of the D1-D2 sequences of the selected strains were conducted using MEGA version 5 [39]. The DNA sequences were aligned with the sequences of homologous regions of closely related species retrieved from GenBank. Evolutionary distances were computed using the Jukes–Cantor method [40] and phylogenetic trees were obtained via neighbor joining [41]. All positions containing alignment gaps and missing data were eliminated in pairwise sequence comparisons (pairwise deletion option). The stability of clades was assessed with 1000 bootstrap replications [42]. The trees are drawn to scale, with branch lengths in the same units as those of the evolutionary distances used to infer the phylogenetic tree. The evolutionary distances were computed using the Jukes–Cantor method [40] and are in the units of the number of base substitutions per site. All positions containing gaps and missing data were eliminated. Evolutionary analyses were conducted using MEGA5 [39].

### 2.6. Effect of Yeast on Botrytis cinerea Spore Germination

The effect of yeast strains selected for their ability to biocontrol grey mold with regard to spore germination were tested in Malt Extract Broth (MEB). Microcentrifuge tubes (1.5 mL) containing 800 μL of MEB were inoculated with 100 μL of a *B. cinerea* conidial suspension (1 × 10^4^ conidia mL^−1^) and 100 μL of yeast suspensions at different concentrations (1 × 10^5^, 1 × 10^6^, 1 × 10^7^, 1 × 10^8^ and 1 × 10^9^ cells mL^−1^). The yeast suspension in the control treatment was substituted with distilled sterile water [43]. Three replicates per treatment were performed. After 24 h at 20 °C, suspensions from each tube were observed microscopically to determine the number of germinated conidia out of a total of 100 conidia. A conidium was considered germinated when the length of the germination tube was more than twice the greatest conidium diameter.

The conidial germination index was calculated from the results according to the formula:

(1) $$\mathrm{Conidial}\;\mathrm{germination}\;\mathrm{index}=\frac{\mathrm{number}\;\mathrm{of}\;\mathrm{germinated}\;\mathrm{conidia}}{\mathrm{total}\;\mathrm{number}\;\mathrm{of}\;\mathrm{conidia}\;\mathrm{counted}}\times \;100$$

### 2.7. Effect of Yeast Concentration on Biocontrol Efficacy of Botrytis cinerea In Vivo

To evaluate the effect of the concentration of the yeast strains selected for their ability to biocontrol grey mold, wounded berries were submerged in a yeast suspension at concentrations of 1 × 10^7^, 1 × 10^8^ or 1 × 10^9^ cells mL^−1^ in accordance with each treatment. Twenty-four hours later, they were sprayed with a conidial suspension of the pathogen at a concentration of 1 × 10^4^ spores mL^−1^.

After 7 days at 20 °C, rot incidence was recorded according to Vero et al. [37]. Three replicates were established for each treatment in a completely randomized design in which each replicate was made up of 28 berries and the entire experiment was repeated twice.

### 2.8. Population Dynamics of Yeasts in the Wounds

The yeast population of yeast strains selected for their ability to biocontrol grey mold was monitored. Disinfected berries was wounded along the equatorial axis, inoculated with 10 μL of yeast suspension at 1 × 10^9^ cells mL^−1^ and stored at 20 °C for 7 days. Tissue samples were taken at different times (0, 1, 2, 3, 4, 5, 6 and 7 days) after treatment; for this, wounded tissue was removed with a crock borer, homogenized in 10 mL of sterile 0.05 mM phosphate buffer (pH 6.5), vortexed, serially diluted and plated in triplicate on YPD. The plates were incubated at 25 °C for 48 h. Colonies were then counted and the results are expressed as the mean number of CFUs per wound [44]. Three replicates were performed for each sampling period, and each replicate was a wound.

### 2.9. Evaluating Oxidative Stress Tolerance

Overnight yeast cultures were washed with sterile distilled water as described above. Ten mL of yeast cell suspension (1 × 10^7^ cell mL^−1^) was exposed to a final concentration of 5 mM H_2_O_2_ at 25 °C on a rotary shaker (100 rpm) for 20, 40 or 60 min. Subsequently, the yeasts were collected, washed and adjusted to a concentration at 1 × 10^3^ cell mL^−1^, of which 100 μL was spread on YPD medium [45]. The plates were incubated at 25 °C for 48 h. Colonies were then counted and the results are expressed as the mean number of CFUs per mL. Three replicates were performed, and the experiment was repeated twice.

### 2.10. Determination of Different Modes of Action for Selected Yeast Strains

#### 2.10.1. Antifungal Activity

Molten sterile YMA medium (0.3% yeast extract, 0.5% malt extract, 0.5% peptone, 1% dextrose, 30 mg L^−1^ methylene blue and 2% agar) adjusted to the pH values of 4.2, 4.6, 5.0 and 5.4 with a citrate–phosphate buffer was inoculated with a conidial suspension of the pathogen to reach a final concentration of 6 × 10^3^ conidia mL^−1^. Fifteen mL of the inoculated medium was dispensed in sterile Petri dishes (9 cm diameter). A yeast aliquot (10 μL) was disposed on the gelled culture medium. The diameter of the inhibition halos of the fungal mycelial growth around the yeast colony was determined after 3–4 days of incubation at 25 °C [46]. Three replicates were performed, and the experiment was repeated three times.

#### 2.10.2. Secretion of β-1,3-Glucanase and Chitinase Activity

The cell wall preparations (CPWs) of *B. cinerea* were obtained with the methodology described by Saligkarias et al. [47]. In minimal YNB medium (0.67% Yeast Nitrogen Base) supplemented with 1 mg mL^−1^ of the fungus wall, the yeast was cultured on a rotary shaker (100 rpm) at 25 °C [46]. Aliquots were taken from the culture 0, 1, 2, 3, 4, 6, 8, 10, 12 and 14 days post-inoculation. These aliquots were centrifuged for 10 min at 3000× *g* and the β-1,3-glucanase and chitinase contents were determined from the supernatant. Production of β-1,3-glucanase was determined by adding 250 μL of supernatant in 250 μL of potassium acetate buffer containing 1% of laminarin. The enzyme–substrate mixture was incubated for 2 h at 40 °C. Then, 0.5 mL of dinitrosalisilic acid reagent was added, followed by boiling at 100 °C for 5 min. After cooling, it was diluted with 5 mL of distilled water and absorbance was measured with a spectrophotometer (Optizen POP BIO, Mecasys Co., Ltd., Daejeon, Republic of Korea) at 595 nm. The protein content of the enzymatic solution was determined in accordance with the method described by Bradford [48] using bovine serum albumin (Sigma A-7906, Sigma-Aldrich, St. Louis, MO, USA) as a standard. Enzymatic activity was expressed as μmol of reducing sugar per milligram protein per hour. Three replicates were performed, and the experiment was repeated twice.

Chitinase production was evaluated by adding 200 μL of supernatant in 1800 μL of p-nitrophenyl N-acetylglucosaminide (Sigma N9376-1G, Sigma-Aldrich, St. Louis, MO, USA). The enzyme–substrate mixture was incubated for 2 h at 40 °C. The reaction was then stopped with 200 μL of NaOH and the absorbance was measured with a spectrophotometer (Optizen POP BIO, Mecasys Co., Ltd., Daejeon, Republic of Korea) at 405 nm. The protein levels were determined with the abovementioned method, and enzymatic activity was expressed as μmol of p-nitrophenol per milligram protein per hour [46]. Three replicates were performed, and the experiment was repeated twice.

#### 2.10.3. Siderophore Production

Siderophore production was determined with CAS agar medium, which was prepared as follows: 30.2 mg of CAS was dissolved in 5 mL of iron (III) solution (1 mM FeCl_3_ in 10 mM HCl). Under stirring, this solution was slowly mixed with hexadecyltrimethyl-ammonium bromide (HDTMA) (36.5 mg of HDTMA dissolved in 20 mL water). The resultant solution was autoclaved and mixed with sterile basal medium (15 g agar, 7.5 g Pipes, 6.7 YNB and 5 g of glucose per L). The yeasts were streaked, and after 48 h of incubation at 25 °C, the detection of siderophores was evidenced by the formation of orange halos around colonies [49]. Three replicates were performed, and the experiment was repeated twice.

#### 2.10.4. Production of Biofilm

Polysterene tissue culture multidishes (Nunclon) with 1800 μL of sterile grape juice were inoculated with a yeast suspension (1 × 10^9^ cells mL^−1^). The yeast suspension in the control treatment was substituted with distilled sterile water. The cells were emptied and washed after 2 days of incubation at 25 °C. The biofilm layer on the wall of the wells was fixed by air-drying and stained with 2 mL of 1% crystal violet for 20 min; the cells were washed and dried again, after which 2 mL of ethanol was added. Absorbance of the eluate was determined at 620 nm with a spectrophotometer (Optizen POP BIO, Mecasys Co., Ltd., Daejeon, Republic of Korea). Biofilm formation was considered to be positive when absorbance was equal to that of the control plus three times the standard deviation [50]. Four replicates were performed, and the experiment was repeated twice.

### 2.11. Data Analysis

The conidial germination index and rot incidence were subjected to an analysis of variance (ANOVA) using the statistical software InfoStat 2016e (FCA-UNC, Córdoba, Argentina); significance was assessed at the 5% significance level (*p* ≤ 0.05) and a Tukey or Student’s *t*-test was used to separate means.

## 3. Results

### 3.1. Obtaining Antagonistic Yeasts

A total of 74 epiphytic yeast strains were isolated from the surface of grapes and 51 from apples; they were maintained in cell suspension with 30% *v*/*v* of glycerol and 70% YPD broth at −20 °C and placed in the microorganism collection of the Faculty of Agronomy at the Universidad de Concepción.

### 3.2. Selecting Yeasts with Antagonistic Activity against Botrytis cinerea

#### 3.2.1. Inhibiting Mycelial Growth of *Botrytis cinerea*

**Figure 1:**
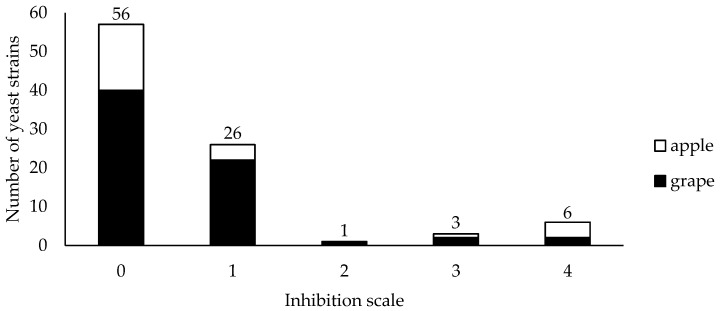
Biocontrol activity of yeasts isolated from grapes 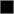 and apples 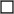 against *Botrytis cinerea* in dual culture on MEA plates at 25 °C. The inhibition of mycelium growth was evaluated in accordance with the scale: 0 = no visible signs of *Botrytis cinerea* inhibition and mycelium surpassed the yeast colony; 1 = both organisms stopped growing on contact; 2 = inhibition zone between pathogen and antagonist was <2 mm; 3 = inhibition zone was between 2 and 4 mm; 4 = inhibition zone was >4 mm.

Of the total strains (*n* = 125), 36 exhibited some degree of antagonistic activity against *B. cinerea* under in vitro conditions, and 10 strains were selected to be tested in vivo since they exhibited inhibition ratings of 2, 3 and 4 on the Swalding and Jeffries [36] scale (Figure 1).

#### 3.2.2. Inhibition of Gray Mold Rot on Table Grapes

**Figure 2:**
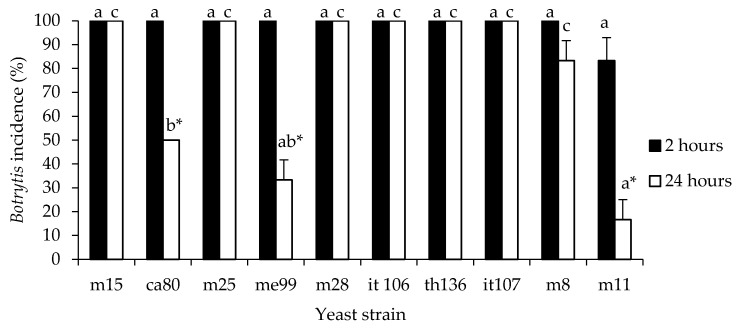
Rot incidence for *Botrytis cinerea* in grapes ‘Thompson seedless’ treated with indigenous yeasts. Fruits were wounded, treated by dipping for 30 s in a yeast strain cell suspension (1 × 10^8^ cells mL^−1^), treated after 2 or 24 h with a spore suspension of fungal pathogen (1 × 10^4^ spores mL^−1^) and stored at 20 °C for 7 days. Different letters indicate significant differences according to the Tukey test (*p* ≤ 0.05). *: indicates that the values differ significantly for the same yeast according to Student’s *t*-test (*p* ≤ 0.05).

Of the 10 yeast strains, 4 exhibited biocontrol activity against *B. cinerea* in grape berries at 20 °C (Figure 2), and the 3 strains m11 (isolated from apple), me99, and ca80 (isolated from grape) reduced gray mold incidence in berries to 16.7, 33.3 and 50.0%, respectively, when the berries were submerged in a yeast suspension at a concentration of 1 × 10^8^ cells mL^−1^ 24 h before applying suspensions of *B. cinerea*. These strains were selected for the next assays.

### 3.3. Identifying Yeasts with Biocontrol Potential against Botrytis cinerea

Comparing restriction fragments obtained from the three yeast strains, two of them exhibited the same pattern (me99 and ca80); when compared with the patterns referenced in previous work [38,51], and the sequences available from the GenBank database, it was determined that strain m11corresponded to *Meyerozyma guilliermondii* (99% similarity with the published sequences for *Pichia guillermondi* Accession No. EF222224.1) and strains me99 and ca80 belonged to the *Aureobasidium pullulans* species (99% similarity with the published sequences for *A. pullulans* Accession No. HM849619.1) (Table 1).

**Figure 3:**
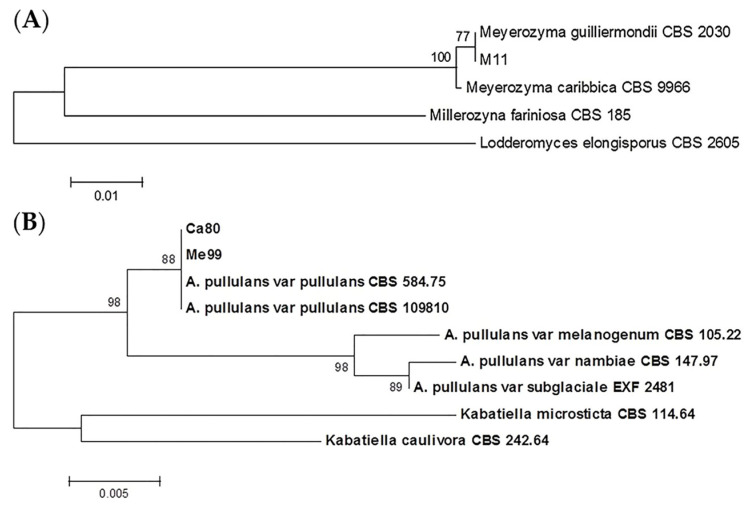
Phylogenetic trees showing placement of selected biocontrol agents m11 and ca80 (**A**) and me99 (**B**) among related species as represented by the optimal trees derived from neighbor joining analysis of 26S rDNA domain D1/D2. *Lodderonyces elongisporus* was used as outgroup species in the first case (**A**) and *Kabatiella microsticta* and *Kabatiella caulivora* were used as outgroup species in the second case (**B**). The percentage of replicate trees in which the associated taxa clustered together in the bootstrap test (1000 replicates) are shown next to the branches [42].

Phylogenetic analysis was performed of the sequences corresponding to the D1/D2 domains of the 26Ss from me99 and ca80 against a dataset of sequences from different *A. pullulans* varieties, placing these strains in the *A. pullulans* var. *pullulans* clade (Figure 3A). D1/D2 sequences from both selected strains showed a 100% homology with the same sequences from *A. pullulans* var. *pullulans* CBS 584.75 and CBS 109,810, as well as differing by 1% of substitutions with the sequence corresponding to *A. pullulans* var. *melanogenum* CBS 105.22, the most closely related variety. In the case of m11, the phylogenetic tree placed it in the same clade as the *M. guilliermondii* CBS 2030 type strain, very closely related to the *Meyerozyma caribbica* (Vaughan-Mart., Kurtzman, S.A. Mey. & E.B. O’Neill) Kurtzman & M. Suzuki type strain (Figure 3B).

### 3.4. Effect of Yeast on Botrytis cinerea Spore Germination

The three yeast strains (m11, me99 and ca80) completely inhibited spore germination of *B. cinerea* at a concentration of 1 × 10^9^ cells mL^−1^ in an MEB medium after 24 h of incubation at 20 °C (Table 2). As yeast concentration increased, conidial germination was inhibited; there was practically no inhibition of germination in all treatments when yeasts were at a concentration of 1 × 10^5^ cells mL^−1^, but the inhibition was significant when the concentration reached 1 × 10^7^ cells mL^−1^.

### 3.5. Effect of Yeast Concentration on Biocontrol Efficacy of Botrytis cinerea in Fruit

**Figure 4:**
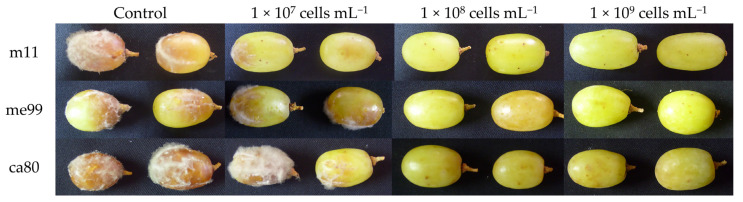
Rot incidence of *Botrytis cinerea* in ‘Thompson Seedless’ grape berries. Fruits were wounded, treated by dipping for 30 s in a cell suspension of yeast strain (m11, me99 and ca80), treated after 24 h with a spore suspension of pathogen (1 × 10^4^ spores mL^−1^) and stored at 20 °C for 7 days.

When yeast concentration increased, rot incidence of *B. cinerea* in table grape berries at 20 °C significantly decreased in the three evaluated strains (Table 3). At a concentration of 1 × 10^9^ cells mL^−1^, the lowest incidence was exhibited by strain m11 (11.9%) (Figure 4).

### 3.6. Population Dynamics of Yeasts in the Wounds

**Figure 5:**
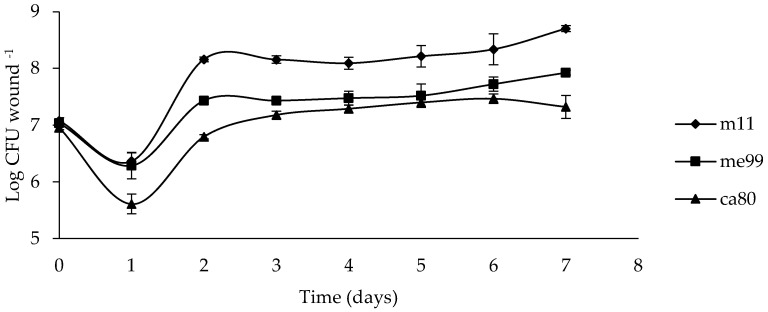
Population dynamics of yeast strains (m11, me99 and ca80) in ‘Thompson Seedless’ grape berry wounds at 20 °C for 7 days. Wounds were treated with 20 μL of cell suspension (1 × 10^9^ cells mL^−1^). Bars represent standard error.

The wound population of yeast during the first day post-inoculation decreased between 0.7 and 1.3 units log UFC for strains m11 and ca80, respectively. On the second day, the strain m11 population increased to 1.8 units log UFC compared to the previous day, while strains me99 and ca80 increased their population to 1 and 1.2 units log UFC, respectively, and afterwards remained practically constant (Figure 5).

### 3.7. Evaluating Oxidative Stress Tolerance

**Figure 6:**
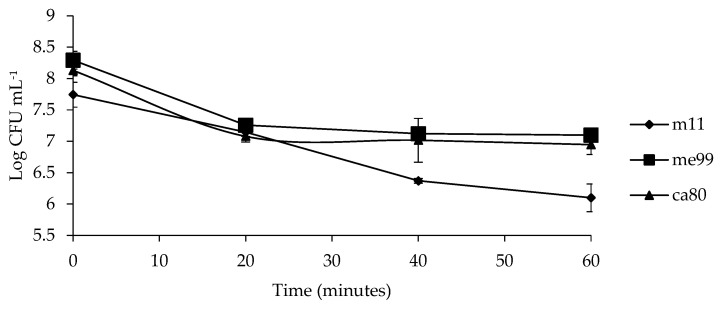
Viability of yeast strains (m11, me99, and ca80) under oxidative stress (5 mM H_2_O_2_) at different exposure times. Bars represent standard error.

The cell viability of the different yeast strains (m11, me99 and ca80) decreased as exposure time to H_2_O_2_ increased (Figure 6). A concentration of 5 mM for 20 min affected all the yeast strains, with a decrease of 0.70, 1.04, and 1.06 units log UFC for strains m11, me99, and ca80, respectively. After 60 min of exposure to H_2_O_2_, strain m11 was the most affected because its population decreased by 1.05 units log UFC compared to the population after 20 min, while strains me99 and ca80 also decreased by 0.16 and 0.13 units log UFC, respectively.

### 3.8. Determination of Yeast Modes of Action

#### 3.8.1. Antifungal Activity

The three strains exhibited antifungal activity against *B. cinerea* at pH 4.6, but only two strains exhibited antifungal activity at pH 4.2 (me99 and ca80) and 5.0 (m11 and ca80), while none of the strains exhibited antifungal activity at pH 5.4 (Table 4).

#### 3.8.2. Secretion of β-1,3-Glucanase and Chitinase Activity

**Figure 7:**
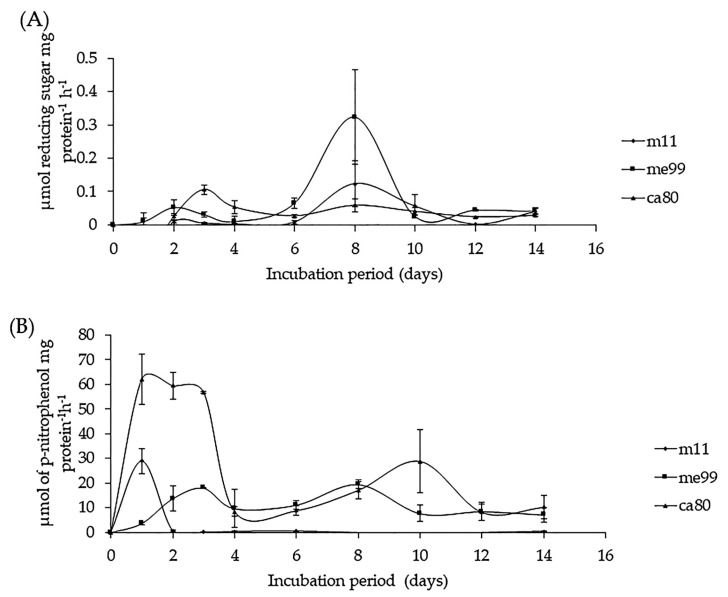
Enzyme activity of yeast strains (m11, me99, and ca80) cultured in a YNB medium supplemented with 1% CWP as sole carbon source at 25 °C. (**A**) β-1,3-glucanase activity and (**B**) chitinase activity. Bars represent standard error.

Enzymatic activity produced against CPWs of the *B. cinerea* fungus, as the only source of carbon, was detected in all three yeast strains. β-1,3-glucanase activity was observed on the first day of incubation, reaching its maximum level on day 3 for strain ca80 and day 8 for strain me99 and strain m11; after this, activity declined in the three yeast strains (Figure 7A). Chitinase activity was observed on the first day of yeast incubation, reaching its maximum level between the first (m11 and ca80) and third (me99) days of incubation, and then decreasing after 48 h of incubation (Figure 7B).

#### 3.8.3. Siderophore Production

**Figure 8:**
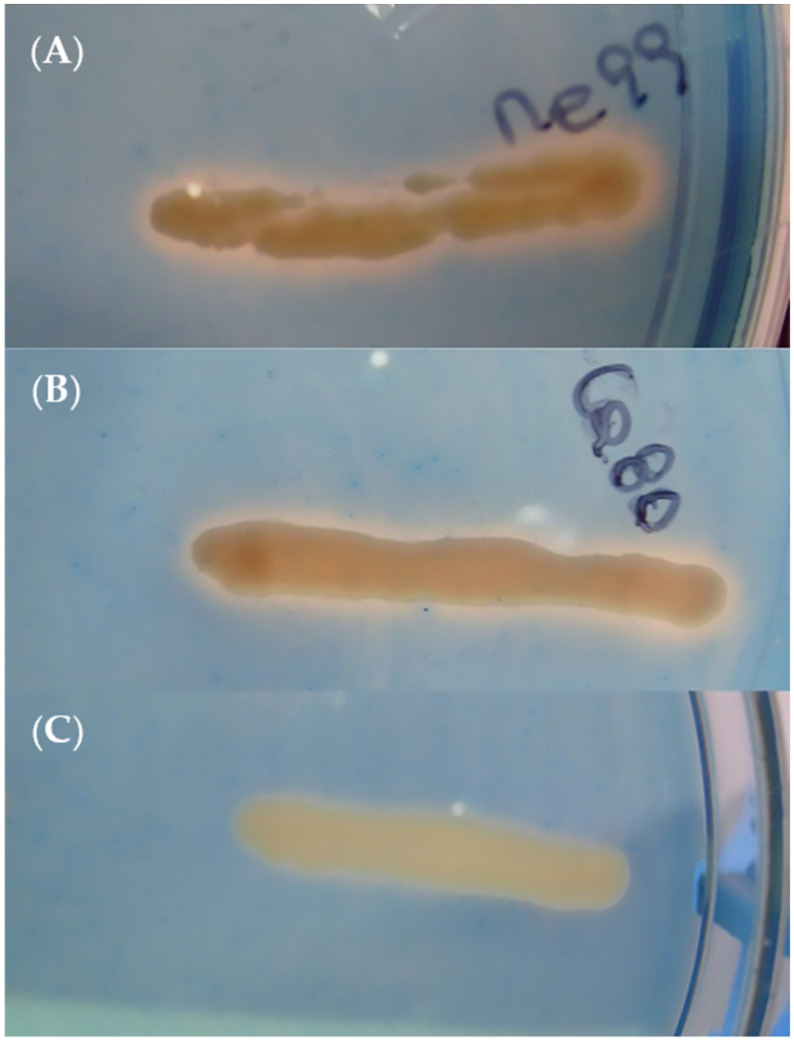
Siderophore production of yeast strains ((**A**) m11, (**B**) me99 and (**C**) ca80) after 48 h of incubation at 25 °C on CAS agar medium.

In the evaluation of siderophore production using CAS media (Table 4), the formation of orange halos around the colonies of strains me99 and ca80 was observed, but not m11; this indicates that only the first two have the ability to produce siderophores (Figure 8).

#### 3.8.4. Production of Biofilm

The ability to produce biofilm in tissue culture multidishes was evaluated as positive for one (m11) of the three evaluated yeast strains, as it exhibited higher absorbance (*A_620_*) than the cutoff criterion (0.064) (Table 5).

## 4. Discussion

This study demonstrated that three indigenous Chilean epiphytic yeast strains (m11, me99 and ca80) isolated from the surface of apples and grapes from commercial orchards and identified as *Meyerozyma guilliermondii* (m11) and *Aureobasidium pullulans* (me99 and ca80) can control gray mold on table grapes.

The use of biocontrol agents to decrease postharvest damage in fruits has been explored as an alternative to synthetic fungicides [12], and the best source of antagonists is the fruit surface [52]. The adopted isolation and selection strategy is crucial in detecting agents with biocontrol potential [53]. An adequate and rapid in vitro preselection strategy is fundamental [36] for evaluating a large number of strains with biocontrol potential [54] against the damage caused by regional strains of the fungal pathogens [55]. In this study, 125 yeast isolates were obtained from the surfaces of table grapes and apples, of which 10 exhibited inhibition ratings of 2, 3 and 4 on the Swalding and Jeffries [35] scale against *B. cinerea* under in vitro conditions. Parafati et al. [53] previously demonstrated that *A. pullulans* was able to inhibit mycelial growth of *B. cinerea* in PDA medium, whereas inhibition was observed in MEA medium in the present study. The pathogen inhibition zones produced in the dual cultures in different solid substrates suggest that the antagonistic yeasts could produce some toxic metabolites for the pathogens under in vitro conditions [14]. However, the production of these toxic metabolites in the culture medium would not necessarily imply its production on the fruit surface [54].

When the 10 yeast isolates selected in vitro were tested on fruit, three isolates (*M. guilliermondii* strain m11 and *A. pullulans* strains me99 and ca80) reduced the incidence of gray mold in grapes when the yeasts were applied 24 h before the pathogen. Previously, Vargas et al. [34] demonstrated that inoculation of biocontrol yeast 24 h before the pathogen significantly reduced the incidence of *B. cinerea* in grapes compared to grapes inoculated with the yeast 1 h before the pathogen. This difference in the reduction of incidence could be explained by the fact that by inoculating the yeast 24 h before the pathogen, the yeast can grow and develop rapidly in the fruit, becoming the dominant microorganism, and the pathogen loses the ability to claim space, so it gradually dies because there is no space [19].

*M. guilliermondii* strain m11 and *A. pullulans* strains me99 and ca80 inhibited conidial germination of *B cinerea* at 20 °C in MEB medium. In fact, at the concentration of 1 × 10^9^ cells mL^−1^, the germination of conidia of *B. cinerea* was completely inhibited by the three strains. The results of this study agree with those obtained by Zhang et al. [56], who reported that at a concentration of 1 × 10^9^ cells mL^−1^ of *M. guilliermondii*, the germination of conidia of *B. cinerea* was completely inhibited and that the germination of conidia increased when the concentration of yeast cells in the medium decreased. Similar results were reported by Zhang et al. [57] with *A. pullulans*, but they reported that at a concentration of 1 × 10^8^ cells mL^−1^, the germination of conidia was only 1.7%.

This antagonistic activity displayed by yeast against the germination of *B. cinerea* conidia could be attributed to competition for nutrients [28], as it has been reported that germination is dependent on the amount of amino acids present in the environment [58]. In this sense, Di Francesco et al. [59] postulated that this inhibition of germination was due to the impediment of nutrient intake of the pathogen caused by the yeast.

In this research, *A. pullulans* strains me99 and ca80 at a concentration of 1 × 10^9^ cells mL^−1^ reduced the incidence of gray mold on table grapes to 26.1 and 32.1%, respectively, and *M. guilliermondii* strain m11 reduced it to 11.9% (Table 3). The control of gray mold by *A. pullulans* and *M. guilliermondii* has been reported by Lima et al. [60], Ippolito et al. [61], Schena et al. [62], Vero et al. [63]), Zhang et al. [56], Mari et al. [14], Parafati et al. [53], Wang et al. [64], Sun et al. [65] and Agarbati et al. [66], but very few studies have been developed on table grapes. Different results have been obtained depending on the strain and the host used.

In this study, when yeast concentration increases, the rot incidence of *B. cinerea* in table grapes decreases significantly; relationships between the concentration of the antagonist and biocontrol efficacy have been described [16,31,54,67,68,69,70,71,72,73,74] because the biocontrol activity of the antagonist is mainly based on the rapid colonization of wounds on the fruit [75].

By comparing the growth of the three yeast strains in the fruit wounds, it could be observed that the population of yeast strains in wounds during the first day post-inoculation decreased (Figure 5). However, *M. guilliermondii* strain m11 rapidly increased in population on the second day, with a higher population compared to those of *A. pullulans* strains me99 and ca80. This ability of *M. guilliermondii* to survive and multiply in wounds has been described by Lahlali et al. [76]. The highest population and rapid colonization in the wounds of *M. guilliermondii* strain m11 could explain the greater control observed against *B. cinerea* since rapid growth and a relatively high population density of microbial antagonists in fruit surface wounds is an advantage in competing with pathogens for nutrients and space and is therefore considered a major mode of action in many postharvest biocontrol systems [77].

To explain the observed decrease in the population of yeast in the first day post-inoculation, the resistance to oxidative stress of yeast strains was studied (Figure 6). Oxidative stress affected the viability of the three yeast strains when exposure time to H_2_O_2_ was increased; however, these yeast strains were able to overcome this stress and finally exceed the initially inoculated population size (Figure 5).

Liu et al. [45] reported a viability of 93% in *Cystofilobasidium infirmominiatum* when it was subjected to a concentration of 5 mM H_2_O_2_ for 20 min, while Hu et al. [73] reported a viability of 80% in *Cryptococcus laurentii* when it was subjected to a concentration of 10 mM H_2_O_2_ for 40 min; these values are higher those for the strains m11, me99, and ca80, which reached survival rates of 17.0, 9.9 and 16.5%, respectively. Antagonists with the ability to produce resistance to this type of stress have an advantage in the control of wound pathogens [78], since the accumulation of reactive oxygen species (ROS) is one of the first signals of a defense reaction to the attack of a pathogen [79]. Therefore, when the yeasts are applied, the ROS generated produce oxidative stress that can affect their viability and behavior [80]. Moreover, necrotrophic pathogens induce oxidative stress or excrete toxins in the host tissue to trigger the cell death process [81]. Necrotic pathogens can take advantage of the initial levels of hydrogen peroxide on the wound to colonize and overcome the plant defense, reducing or even eliminating peroxide levels generated by the fruit [82].

The three strains exhibited antifungal activity against *B. cinerea* at pH 4.6, although not at pH 5.4. Similar results were also obtained by Parafati et al. [53] and Grzegorczyk et al. [32] where acidic conditions emphasized this antagonism, which could be related to the production of killer toxins whose action and production depend on pH [46]; these toxins are inactive at a pH over 5 [83].

The enzymatic activities of the chitinase and β-1,3-glucanase types produced by *M. guilliermondii* against the CPW of *B. cinerea* fungus have been reported in previous studies [18,56,57], but in the case of strain m11, the maximum-level chitinase activity was attained after 24 h of incubation, while maximum activity was attained after 48 h for the strain reported by Zhang et al. [56]. This difference was also seen in the activity of β-1,3-glucanase because strain m11 exhibited its maximum level on day 8, while this was reached after 96 h of incubation in the strain described by Zhang et al. [56]. Vero et al. [63] reported that *A. pullulans* at 5 °C have β-1,3-glucanase activity starting after 7 days of incubation with CPWs of *Penicillium expansum* as the sole carbon source, exhibiting the maximum level of activity on day 25, while the maximum level chitinase activity was after 30 days.

The production of β-1,3- glucanase and chitinase during the early stages of yeast growth suggests that these enzymes play a role in breaking down complex polysaccharide polymers, which can then be consumed as a carbon source by the yeast cells [84]. β-1,3-glucanase acts on the β-1,3-glucan sites in the polysaccharide chain of the pathogen hyphal walls, distorting the hyphae and leaking their cytoplasmic components [85], thereby causes cell lysis and subsequent death.

Siderophore production was only detected in *A. pullulans* strains me99 and ca80, a feature that has been demonstrated in marine strains of *A. pullulans* [86]. This yeast species has been described as producing hydroxamate-type molecules that can act as siderophores when they grow in a medium containing a low concentration of iron [87]. Grape berries contain few iron ions [88], so the evaluated yeast strains should be able to produce siderophores in the wound, leaving the iron unavailable; this element is essential to the conidial germination of *B. cinerea* [89].

One of the three yeast strains (m11) exhibited the ability to produce biofilms; this ability has been suggested as a mode of action used by *Cryptococcus victoriae*, *Debaryomyces nepalensis*, *M. pulcherrima*, *Pichia membranaefaciens* and *W. anomalus* [21,23,25,43,53].

In fact, biofilm production favors the attachment, colonization and growth of antagonists on the surface of fruits, enhancing the ability of colonizing microorganisms to obtain nutrients [23]. This feature depends on the ability of the cells to adhere to different surfaces; this adhesion is caused by specialized proteins called adhesines that unite with amino acids and sugar residues from the surfaces of other cells or abiotic surfaces [90], creating a mechanical barrier that stands between the wound and the pathogen [80].

These results suggest that *M. guilliermondii* strain m11 and *A. pullulans* strains me99 and ca80 have potential as biocontrol agents of *B. cinerea* in ‘Thompson seedless’ table grapes. In addition, they suggest the possibility that chitinase and β-1,3-glucanase activity, siderophore production, or the ability to produce biofilms might be involved in the biocontrol efficacy of antagonistic yeasts.

## Figures and Tables

**Table 1:** Size in base pairs (bp) of 5.8S-ITS PCR products and ribosomal DNA restriction patterns for yeast strains after digestion with the restriction endonucleases *HaeIII*, *HinfI* and *HhaI*.

Yeast Strains	PCR Product ITS1-ITS4	Restriction Fragments			Species
		*HaeIII*	*HinfI*	*HhaI*	
m11	600	400 + 115 + 90	320 + 300	300 + 265 + 60	*Meyerozyma guilliermondii* ^1^
me99	590	460 + 150	290 + 180 + 130	190 + 180 + 100	*Aureobasidium pullulans* ^2^
ca80	590	460 + 150	290 + 180 + 130	190 + 180 + 100	*Aureobasidium pullulans* ^2^

^1^: according to Esteve-Zarzoso et al. [38]; ^2^: according to Sabate et al. [51]. The percentage identity of 5.8S-ITS ribosomal DNA was calculated with BLAST program and the sequences were compared with those from NCBI database (www.ncbi.nlm.nih.gov, accessed on 1 January 2020).

**Table 2:** Effect of yeast strains m11, me99 and ca80 on spore germination of *Botrytis cinerea* by co-culturing in MEB at 20 °C for 24 h.

Concentration of Yeast (Cells mL^−1^)	Conidial Germination Index (%) *		
	m11	me99	ca80
Control	100.00 ± 0.00 ^a^	100.00 ± 0.00 ^a^	100.00 ± 0.00 ^a^
1 × 10^5^	99.67 ± 0.33 ^a^	100.00 ± 0.00 ^a^	97.67 ± 0.67 ^a^
1 × 10^6^	92.67± 3.28 ^a^	90.00 ± 2.65 ^b^	96.33 ± 0.67 ^a^
1 × 10^7^	18.00± 3.00 ^b^	23.33 ± 3.48 ^c^	19.67 ± 2.33 ^b^
1 × 10^8^	3.67 ± 1.33 ^c^	6.67 ± 0.88 ^d^	0.33 ± 0.33 ^c^
1 × 10^9^	0.00 ± 0.00 ^c^	0.00 ± 0.00 ^d^	0.00 ± 0.00 ^c^

Data expressed as mean ± standard error. *: conidial germination index = (number of germinated conidia/total number of conidia counted) × 100. Control treatment means the yeast suspension was substituted with distilled sterile water. Different letters in the same column indicate differences among concentrations according to the Tukey test (*p* ≤ 0.05).

**Table 3:** Effect of different concentrations of yeast strains m11, me99 and ca80 on biocontrol efficacy of *Botrytis cinerea* in ‘Thompson Seedless’ grape berries. Fruits were wounded, treated by dipping for 30 s in a cell suspension of yeast strain, treated after 24 h with a spore suspension of pathogen (1 × 10^4^ spores mL^−1^) and stored at 20 °C for 7 days.

Concentration of Yeast (Cells mL^−1^)	Rot Incidence (%)		
	m11	me99	ca80
Control	78.81 ± 4.76 ^c^	84.52 ± 6.63 ^b^	94.05 ± 1.19 ^c^
1 × 10^7^	34.52 ± 7.81 ^b^	64.29 ± 5.46 ^b^	60.71 ± 7.14 ^b^
1 × 10^8^	20.24 ± 4.76 ^ab^	35.71 ± 2.06 ^a^	33.33 ± 5.19 ^a^
1 × 10^9^	11.90 ± 6.30 ^a^	26.19 ± 7.81 ^a^	32.14 ± 2.06 ^a^

Data expressed as mean ± standard error. Control means the treatment of pathogen only. Different letters in the same column indicate differences among concentrations according to the Tukey test (*p* ≤ 0.05).

**Table 4:** Antifungal activity of yeast strains (m11, me99 and ca80) against *Botrytis cinerea* on YMA plates at different pH.

Yeast Strain	pH			
	4.2	4.6	5.0	5.4
Control *	−	−	−	−
m11	−	+	+	−
me99	+	+	−	−
ca80	+	+	+	−

*: control consisted of *Botrytis cinerea* incorporated into the medium without yeast. +: presence of inhibition halo. −: absence of inhibition halo.

**Table 5:** Siderophore production in CAS medium and biofilm formation in tissue culture multidishes by yeasts at 25 °C.

Yeast Strain	Siderophore Production	Biofilm Formation	
		Absorbance (A_620_)	
m11	−	0.076 ± 0.006	+
me99	+	0.008 ± 0.002	−
ca80	+	0.010 ± 0.001	−

+: presence of siderophores or biofilm. −: absence of siderophores or biofilm. Cutoff value of biofilm formation = 0.064. Biofilm formation data are expressed as mean ± standard error.

## Data Availability

Not applicable.

## References

[1] Esterio M., Osorio-Navarro C., Azócar M., Copier C., Rubilar M., Pizarro L., Auger J. (2021). Reduced fitness cost and increased aggressiveness in fenhexamid-resistant *Botrytis cinerea* field isolates from Chile. Phytopathol. Mediterr..

[2] Agrios G.N. (2005). Plant Pathology.

[3] Holz G., Coertze S., Williamson B., Elad Y., Williamson B., Tudzynski P., Delen N. (2007). The Ecology of *Botrytis* on Plant Surfaces. Botrytis: Biology, Pathology and Control.

[4] Latorre B.A., Elfar K., Ferrada E.E. (2015). Gray mold caused by *Botrytis cinerea* limits grape production in Chile. Cienc. Investig. Agrar..

[5] Latorre B., Lillo C., Rioja M. (2001). Effect of timing on the efficacy of fungicide treatments applied against *Botrytis cinerea* of gapevine. Cienc. Investig. Agrar..

[6] Latorre B.A. (1994). Dicarboximide-Resistant Isolates of *Botrytis cinérea* from Table Grape in Chile: Survey and Characterization. Plant Dis..

[7] Esterio M., Auger J., Ramos C., García H. (2007). First Report of Fenhexamid Resistant Isolates of *Botrytis cinerea* on Grapevine in Chile. Plant Dis..

[8] Esterio M., Muñoz G., Ramos C., Cofré G., Estévez R., Salinas A., Auger J. (2011). Characterization of *Botrytis cinerea* Isolates Present in Thompson Seedless Table Grapes in the Central Valley of Chile. Plant Dis..

[9] Piqueras C.M., Herrera D., Latorre B.A. (2014). First Report of High Boscalid Resistance in *Botrytis cinerea* Associated with the H272L Mutation in Grapevine in Chile. Plant Dis..

[10] Esterio M., Araneda M.J., Román A., Pizarro L., Copier C., Auger J. (2015). First Report of Boscalid Resistant *Botrytis cinerea* Isolates Carrying the Mutations H272R, H272Y, P225L, and P225H from Table Grape in Chile. Plant Dis..

[11] Zhang H., Godana E.A., Sui Y., Yang Q., Zhang X., Zhao L. (2020). Biological control as an alternative to synthetic fungicides for the management of grey and blue mould diseases of table grapes: A review. Crit. Rev. Microbiol..

[12] Spadaro D., Droby S. (2016). Development of biocontrol products for postharvest diseases of fruit: The importance of elucidating the mechanisms of action of yeast antagonists. Trends Food Sci. Technol..

[13] Li Q., Li C., Li P., Zhang H., Zhang X., Zheng X., Yang Q., Apaliya M.T., Boateng N.A.S., Sun Y. (2017). The biocontrol effect of *Sporidiobolus pararoseus* Y16 against postharvest diseases in table grapes caused by *Aspergillus niger* and the possible mechanisms involved. Biol. Control.

[14] Mari M., Martini C., Spadoni A., Rouissi W., Bertolini P. (2012). Biocontrol of apple postharvest decay by *Aureobasidium pullulans*. Postharvest Biol. Technol..

[15] Zhang X., Wu F., Gu N., Yan X., Wang K., Dhanasekaran S., Gu X., Zhao L., Zhang H. (2020). Postharvest biological control of Rhizopus rot and the mechanisms involved in induced disease resistance of peaches by *Pichia membranefaciens*. Postharvest Biol. Technol..

[16] Zhao L., Lan C., Tang X., Li B., Zhang X., Gu X., Zhang H. (2022). Efficacy of *Debaryomyce hansenii* in the biocontrol for postharvest soft rot of strawberry and investigation of the physiological mechanisms involved. Biol. Control.

[17] Wilson C.L., Wisniewski M.E. (1989). Biological Control of Postharvest Diseases of Fruits and Vegetables: An Emerging Technology. Annu. Rev. Phytopathol..

[18] Chanchaichaovivat A., Panijpan B., Ruenwongsa P. (2008). Putative modes of action of *Pichia guilliermondii* strain R13 in controlling chilli anthracnose after harvest. Biol. Control.

[19] Tian Y.-Q., Li W., Jiang Z.-T., Jing M.-M., Shao Y.-Z. (2018). The preservation effect of *Metschnikowia pulcherrima* yeast on anthracnose of postharvest mango fruits and the possible mechanism. Food Sci. Biotechnol..

[20] Chan Z., Tian S. (2006). Induction of H_2_O_2_-metabolizing enzymes and total protein synthesis by antagonistic yeast and salicylic acid in harvested sweet cherry fruit. Postharvest Biol. Technol..

[21] Zhou Y., Li W., Zeng J., Shao Y. (2018). Mechanisms of action of the yeast *Debaryomyces nepalensis* for control of the pathogen Colletotrichum gloeosporioides in mango fruit. Biol. Control.

[22] Madbouly A.K., Elyousr K.A.A., Ismail I.M. (2020). Biocontrol of *Monilinia fructigena*, causal agent of brown rot of apple fruit, by using endophytic yeasts. Biol. Control.

[23] Yang H., Wang L., Li S., Gao X., Wu N., Zhao Y., Sun W. (2021). Control of postharvest grey spot rot of loquat fruit with *Metschnikowia pulcherrima* E1 and potential mechanisms of action. Biol. Control.

[24] Parafati L., Restuccia C., Cirvilleri G. (2022). Efficacy and mechanism of action of food isolated yeasts in the control of *Aspergillus flavus* growth on pistachio nuts. Food Microbiol..

[25] Liu Y., Yao S., Deng L., Ming J., Zeng K. (2019). Different mechanisms of action of isolated epiphytic yeasts against *Penicillium digitatum* and *Penicillium italicum* on citrus fruit. Postharvest Biol. Technol..

[26] Di Francesco A., Mari M., Ugolini L., Baraldi E. (2018). Effect of *Aureobasidium pullulans* strains against *Botrytis cinerea* on kiwifruit during storage and on fruit nutritional composition. Food Microbiol..

[27] Gao Z., Zhang R., Xiong B. (2021). Management of postharvest diseases of kiwifruit with a combination of the biocontrol yeast *Candida oleophila* and an oligogalacturonide. Biol. Control.

[28] Alimadadi N., Pourvali Z., Nasr S., Fazeli S.A.S. (2023). Screening of antagonistic yeast strains for postharvest control of *Penicillium expansum* causing blue mold decay in table grape. Fungal Biol..

[29] Zhu Y., Zong Y., Gong D., Zhang X., Oyom W., Yu L., Wang X., Bi Y., Prusky D. (2022). Effects and possible modes of action of *Kloeckera apiculata* for controlling *Penicillium expansum* in apples. Biol. Control.

[30] Ruiz-Moyano S., Martín A., Villalobos M., Calle A., Serradilla M., Córdoba M., Hernández A. (2016). Yeasts isolated from figs (*Ficus carica* L.) as biocontrol agents of postharvest fruit diseases. Food Microbiol..

[31] Shen H., Wei Y., Wang X., Xu C., Shao X. (2019). The marine yeast *Sporidiobolus pararoseus* ZMY-1 has antagonistic properties against *Botrytis cinerea* in vitro and in strawberry fruit. Postharvest Biol. Technol..

[32] Grzegorczyk M., Żarowska B., Restuccia C., Cirvilleri G. (2017). Postharvest biocontrol ability of killer yeasts against *Monilinia fructigena* and *Monilinia fructicola* on stone fruit. Food Microbiol..

[33] Zhang X., Li B., Zhang Z., Chen Y., Tian S. (2020). Antagonistic Yeasts: A Promising Alternative to Chemical Fungicides for Controlling Postharvest Decay of Fruit. J. Fungi.

[34] Vargas M., Garrido F., Zapata N., Tapia M. (2012). Isolation and Selection of Epiphytic Yeat for Biocontrol of *Botrytis cinerea* Pers. on Table Grapes. Chil. J. Agric. Res..

[35] Rabosto X., Carrau M., Paz A., Boido E., Dellacassa E., Carrau F.M. (2006). Grapes and Vineyard Soils as Sources of Microorganisms for Biological Control of *Botrytis cinerea*. Am. J. Enol. Vitic..

[36] Swadling I.R., Jeffries P. (1996). Isolation of Microbial Antagonists for Biocontrol of Grey Mould Disease of Strawberries. Biocontrol Sci. Technol..

[37] Vero S., Mondino P., Burgueño J., Soubes M., Wisniewski M. (2002). Characterization of biocontrol activity of two yeast strains from Uruguay against blue mold of apple. Postharvest Biol. Technol..

[38] Esteve-Zarzoso B., Belloch C., Uruburu F., Querol A. (1999). Identification of yeasts by RFLP analysis of the 5.8S rRNA gene and the two ribosomal internal transcribed spacers. Int. J. Syst. Evol. Microbiol..

[39] Tamura K., Peterson D., Peterson N., Stecher G., Nei M., Kumar S. (2011). MEGA5: Molecular Evolutionary Genetics Analysis Using Maximum Likelihood, Evolutionary Distance, and Maximum Parsimony Methods. Mol. Biol. Evol..

[40] Jukes T.H., Cantor C.R. (1969). Evolution of Protein Molecules. Mammalian Protein Metabolism.

[41] Saitou N., Nei M. (1987). The neighbor-joining method: A new method for reconstructing phylogenetic trees. Mol. Biol. Evol..

[42] Felsenstein J. (1985). Phylogenies and the Comparative Method. Am. Nat..

[43] Lutz M.C., Lopes C.A., Rodriguez M.E., Sosa M.C., Sangorrín M.P. (2013). Efficacy and putative mode of action of native and commercial antagonistic yeasts against postharvest pathogens of pear. Int. J. Food Microbiol..

[44] Bouzerda L., Boubaker H., Boudyach E.H., Akhayat O., Ait Ben Aoumar A. (2003). Selection of Antagonistic Yeasts to Greend Mold Disease of Citrus in Morocco. J. Food Agric. Environ..

[45] Liu J., Wisniewski M., Droby S., Vero S., Tian S., Hershkovitz V. (2011). Glycine betaine improves oxidative stress tolerance and biocontrol efficacy of the antagonistic yeast *Cystofilobasidium infirmominiatum*. Int. J. Food Microbiol..

[46] Hernández-Montiel L.G., Larralde-Corona C.P., Vero S., López-Aburto M.G., Ochoa J.L., Ascencio-Valle F. (2010). Caracterización de levaduras *Debaryomyces hansenii* para el control biológico de la podredumbre azul del limón mexicano Characterization of yeast *Debaryomyces hansenii* for the biological control of blue mold decay of Mexican lemon. CyTA-J. Food.

[47] Saligkarias I., Gravanis F., Epton H. (2002). Biological control of *Botrytis cinerea* on tomato plants by the use of epiphytic yeasts *Candida guilliermondii* strains 101 and US 7 and *Candida oleophila* strain I-182: I. in vivo studies. Biol. Control.

[48] Bradford M.M. (1976). A rapid and sensitive method for the quantitation of microgram quantities of protein utilizing the principle of protein-dye binding. Anal. Biochem..

[49] Shin S.H., Lim Y., Lee S.E., Yang N.W., Rhee J.H. (2001). CAS agar diffusion assay for the measurement of siderophores in biological fluids. J. Microbiol. Methods.

[50] Růžička F., Holá V., Votava M., Tejkalová R. (2007). Importance of biofilm in *Candida parapsilosis* and evaluation of its susceptibility to antifungal agents by colorimetric method. Folia Microbiol..

[51] Sabate J., Cano J., Esteve-Zarzoso B., Guillamon J. (2002). Isolation and identification of yeasts associated with vineyard and winery by RFLP analysis of ribosomal genes and mitochondrial DNA. Microbiol. Res..

[52] Lima G., Sanzani S., De Curtis F., Ippolito A., Wills R., Golding J. (2015). Biological Control of Postharvest Diseases. Advances in Postharvest Fruit and Vegetable Technology.

[53] Parafati L., Vitale A., Restuccia C., Cirvilleri G. (2015). Biocontrol ability and action mechanism of food-isolated yeast strains against *Botrytis cinerea* causing post-harvest bunch rot of table grape. Food Microbiol..

[54] Nally M.C., Pesce V.M., Maturano Y.P., Muñoz C.J., Combina M., Toro M.E., de Figueroa L.C., Vazquez F. (2012). Biocontrol of *Botrytis cinerea* in table grapes by non-pathogenic indigenous Saccharomyces cerevisiae yeasts isolated from viticultural environments in Argentina. Postharvest Biol. Technol..

[55] Robiglio A., Sosa M.C., Lutz M.C., Lopes C.A., Sangorrín M.P. (2011). Yeast Biocontrol of Fungal Spoilage of Pears Stored at Low Temperature. Int. J. Food Microbiol..

[56] Zhang D., Spadaro D., Garibaldi A., Gullino M.L. (2011). Potential biocontrol activity of a strain of *Pichia guilliermondii* against grey mold of apples and its possible modes of action. Biol. Control.

[57] Zhang D., Spadaro D., Garibaldi A., Gullino M.L. (2010). Efficacy of the antagonist *Aureobasidium pullulans* PL5 against postharvest pathogens of peach, apple and plum and its modes of action. Biol. Control.

[58] Janisiewicz W.J., Tworkoski T.J., Sharer C. (2000). Characterizing the Mechanism of Biological Control of Postharvest Diseases on Fruits with a Simple Method to Study Competition for Nutrients. Phytopathology.

[59] Di Francesco A., Ugolini L., D’Aquino S., Pagnotta E., Mari M. (2017). Biocontrol of *Monilinia laxa* by *Aureobasidium pullulans* strains: Insights on competition for nutrients and space. Int. J. Food Microbiol..

[60] Lima G., Ippolito A., Nigro F., Salerno M. (1997). Effectiveness of *Aureobasidium pullulans* and *Candida oleophila* against postharvest strawberry rots. Postharvest Biol. Technol..

[61] Ippolito A., El Ghaouth A., Wilson C.L., Wisniewski M. (2000). Control of postharvest decay of apple fruit by *Aureobasidium pullulans* and induction of defense responses. Postharvest Biol. Technol..

[62] Schena L., Nigro F., Pentimone I., Ligorio A., Ippolito A. (2003). Control of postharvest rots of sweet cherries and table grapes with endophytic isolates of *Aureobasidium pullulans*. Postharvest Biol. Technol..

[63] Vero S., Garmendia G., González M.B., Garat M.F., Wisniewski M. (2009). *Aureobasidium pullulans* as a biocontrol agent of postharvest pathogens of apples in Uruguay. Biocontrol Sci. Technol..

[64] Wang X., Glawe D.A., Kramer E., Weller D., Okubara P.A. (2018). Biological Control of *Botrytis cinerea*: Interactions with Native Vineyard Yeasts from Washington State. Phytopathology.

[65] Sun C., Huang Y., Lian S., Saleem M., Li B., Wang C. (2021). Improving the biocontrol efficacy of *Meyerozyma guilliermondii* Y-1 with melatonin against postharvest gray mold in apple fruit. Postharvest Biol. Technol..

[66] Agarbati A., Canonico L., Pecci T., Romanazzi G., Ciani M., Comitini F. (2022). Biocontrol of Non-*Saccharomyces* Yeasts in Vineyard against the Gray Mold Disease Agent *Botrytis cinerea*. Microorganisms.

[67] Spadaro D., Vola R., Piano S., Gullino M.L. (2002). Mechanisms of action and efficacy of four isolates of the yeast *Metschnikowia pulcherrima* active against postharvest pathogens on apples. Postharvest Biol. Technol..

[68] Long C.-A., Wu Z., Deng B.-X. (2005). Biological control of *Penicillium italicum* of Citrus and *Botrytis cinerea* of Grape by Strain 34–9 of *Kloeckera apiculata*. Eur. Food Res. Technol..

[69] Zhang H., Wang L., Dong Y., Jiang S., Cao J., Meng R. (2007). Postharvest biological control of gray mold decay of strawberry with *Rhodotorula glutinis*. Biol. Control.

[70] Liu H.M., Guo J.H., Cheng Y.J., Luo L., Liu P., Wang B.Q., Deng B.X., Long C.A. (2010). Control of gray mold of grape by *Hanseniaspora uvarum* and its effects on postharvest quality parameters. Ann. Microbiol..

[71] Manso T., Nunes C. (2011). *Metschnikowia andauensis* as a new biocontrol agent of fruit postharvest diseases. Postharvest Biol. Technol..

[72] Li W., Zhang H., Li P., Apaliya M.T., Yang Q., Peng Y., Zhang X. (2016). Biocontrol of postharvest green mold of oranges by *Hanseniaspora uvarum* Y3 in combination with phosphatidylcholine. Biol. Control.

[73] Hu H., Wisniewski M.E., Abdelfattah A., Zheng X. (2017). Biocontrol activity of a cold-adapted yeast from Tibet against gray mold in cherry tomato and its action mechanism. Extremophiles.

[74] Hernandez-Montiel L.G., Gutierrez-Perez E.D., Murillo-Amador B., Vero S., Chiquito-Contreras R.G., Rincon-Enriquez G. (2018). Mechanisms employed by *Debaryomyces hansenii* in biological control of anthracnose disease on papaya fruit. Postharvest Biol. Technol..

[75] Wang K., Cao S., Rui H., Zheng Y., Jin P. (2011). Biological Control of Green Mould Decay in Postharvest Chinese Bayberries by *Pichia membranaefaciens*. J. Phytopathol..

[76] Lahlali R., Hamadi Y., El Guilli M., Jijakli M.H. (2011). Efficacy assessment of *Pichia guilliermondii* strain Z1, a new biocontrol agent, against citrus blue mould in Morocco under the influence of temperature and relative humidity. Biol. Control.

[77] Tang J., Liu Y., Li H., Wang L., Huang K., Chen Z. (2015). Combining an antagonistic yeast with harpin treatment to control postharvest decay of kiwifruit. Biol. Control.

[78] Sui Y., Wang Z., Zhang D., Wang Q. (2021). Oxidative stress adaptation of the antagonistic yeast, *Debaryomyces hansenii*, increases fitness in the microenvironment of kiwifruit wound and biocontrol efficacy against postharvest diseases. Biol. Control.

[79] Liu J., Sui Y., Wisniewski M., Droby S., Liu Y. (2013). Utilization of antagonistic yeasts to manage postharvest fungal diseases of fruit. Int. J. Food Microbiol..

[80] Di Francesco A., Martini C., Mari M. (2016). Biological control of postharvest diseases by microbial antagonists: How many mechanisms of action?. Eur. J. Plant Pathol..

[81] Yu T., Zhang H., Li X., Zheng X. (2008). Biocontrol of *Botrytis cinerea* in apple fruit by *Cryptococcus laurentii* and indole-3-acetic acid. Biol. Control.

[82] Torres R., Teixidó N., Usall J., Abadias M., Mir N., Larrigaudiere C., Viñas I. (2011). Anti-oxidant activity of oranges after infection with the pathogen *Penicillium digitatum* or treatment with the biocontrol agent *Pantoea agglomerans* CPA-2. Biol. Control.

[83] Santos A., Sánchez A., Marquina D. (2004). Yeasts as biological agents to control *Botrytis cinerea*. Microbiol. Res..

[84] Bar-Shimon M., Yehuda H., Cohen L., Weiss B., Kobeshnikov A., Daus A., Goldway M., Wisniewski M., Droby S. (2004). Characterization of extracellular lytic enzymes produced by the yeast biocontrol agent *Candida oleophila*. Curr. Genet..

[85] Delali K.I., Chen O., Wang W., Yi L., Deng L., Zeng K. (2021). Evaluation of yeast isolates from kimchi with antagonistic activity against green mold in citrus and elucidating the action mechanisms of three yeast: *P. kudriavzevii*, *K. marxianus*, and *Y. lipolytica*. Postharvest Biol. Technol..

[86] Wang W., Chi Z., Li J., Wang X. (2009). Siderophore production by the marine-derived *Aureobasidium pullulans* and its antimicrobial activity. Bioresour. Technol..

[87] Saravanakumar D., Ciavorella A., Spadaro D., Garibaldi A., Gullino M.L. (2008). *Metschnikowia pulcherrima* strain MACH1 outcompetes *Botrytis cinerea*, *Alternaria alternata* and *Penicillium expansum* in apples through iron depletion. Postharvest Biol. Technol..

[88] Nally M., Pesce V., Maturano Y., Assaf L.R., Toro M., de Figueroa L.C., Vazquez F. (2015). Antifungal modes of action of Saccharomyces and other biocontrol yeasts against fungi isolated from sour and grey rots. Int. J. Food Microbiol..

[89] Sansone G., Rezza I., Calvente V., Benuzzi D., de Tosetti M.I.S. (2005). Control of *Botrytis cinerea* strains resistant to iprodione in apple with rhodotorulic acid and yeasts. Postharvest Biol. Technol..

[90] Verstrepen K.J., Klis F.M. (2006). Flocculation, adhesion and biofilm formation in yeasts. Mol. Microbiol..

